# New Kids on the Block: RNA-Based Influenza Virus Vaccines

**DOI:** 10.3390/vaccines6020020

**Published:** 2018-04-01

**Authors:** Francesco Berlanda Scorza, Norbert Pardi

**Affiliations:** 1 PATH’s Center for Vaccine Innovation and Access, 455 Massachusetts Ave. NW, Suite 1000, Washington, DC 20001, USA; fberlanda-scorza@path.org; 2 Department of Medicine, University of Pennsylvania, Philadelphia, PA 19104, USA

**Keywords:** RNA vaccine, influenza virus, infectious disease, clinical trial

## Abstract

RNA-based immunization strategies have emerged as promising alternatives to conventional vaccine approaches. A substantial body of published work demonstrates that RNA vaccines can elicit potent, protective immune responses against various pathogens. Consonant with its huge impact on public health, influenza virus is one of the best studied targets of RNA vaccine research. Currently licensed influenza vaccines show variable levels of protection against seasonal influenza virus strains but are inadequate against drifted and pandemic viruses. In recent years, several types of RNA vaccines demonstrated efficacy against influenza virus infections in preclinical models. Additionally, comparative studies demonstrated the superiority of some RNA vaccines over the currently used inactivated influenza virus vaccines in animal models. Based on these promising preclinical results, clinical trials have been initiated and should provide valuable information about the translatability of the impressive preclinical data to humans. This review briefly describes RNA-based vaccination strategies, summarizes published preclinical and clinical data, highlights the roadblocks that need to be overcome for clinical applications, discusses the landscape of industrial development, and shares the authors’ personal perspectives about the future of RNA-based influenza virus vaccines.

## 1. Introduction

Influenza virus causes millions of illnesses and up to 650,000 deaths worldwide every year [1]. Approved seasonal influenza vaccines protect from well-matched circulating strains but are not effective against drifted seasonal and pandemic viruses; thus, there is an urgent need for more broadly protective vaccines. Recent publications have discussed the challenges that need to be resolved by a broadly protective or universal influenza virus vaccine, such as: lack of protection from antigenically drifted or shifted viral strains, suboptimal epitope-specific antibody responses caused by pre-existing host immunity (original antigenic sin), short-lived protective immunity after vaccine administration, potential side effects of live virus vaccines, or inhibitory effects of maternal antibodies on vaccine immunogenicity in infants [2,3,4,5,6]. The data available to date suggest that RNA-based influenza vaccines have the potential to induce broadly protective immune responses and may address some of the above-mentioned challenges.

Vaccination with in vitro transcribed (IVT) messenger RNA (mRNA) was not widely considered as a viable approach until the early 2000s; however, several early studies using various types of mRNA vaccines demonstrated promising preclinical results [7,8,9]. Seminal publications from the past years made it clear that mRNA vaccines—targeting cancer and infectious diseases—have significant therapeutic potential and might represent the next generation of prophylactic and therapeutic vaccines (reviewed in [10]). 

Early studies using naked (uncomplexed) unmodified RNA for immunization via the intramuscular route demonstrated some efficacy against various infectious pathogens [11] but substantial improvements were necessary to make mRNA into a potent vaccine. The major goals were to increase the in vivo half-life of mRNA and achieve high in vivo translatability to produce large amounts of immunogen for extended periods of time after vaccine administration. Apart from intranodally administered naked mRNA vaccines (discussed in [10]), most potent iterations of directly injectable mRNA vaccines have two major components: (1) a fairly stable, highly translatable, optimized mRNA and (2) a carrier molecule that encapsulates mRNA. (1) mRNA is transcribed by a bacteriophage RNA polymerase from a linear DNA template (linearized plasmid or PCR product) containing a T3, T7 or Sp6 phage promoter [12]. Introduction of naturally occurring modified nucleosides, optimization of the codon composition (replacing rare codons with frequently used synonymous codons) and purification of the in vitro transcribed mRNA can yield increases of many orders of magnitude in protein translation from mRNA via the silencing of various innate immune sensing pathways [13,14,15,16,17,18]. Further optimization steps, such as the incorporation of 5′ and 3′ UTR elements that increase mRNA stability and translatability, optimization of the length of poly(A) tail and addition of a 5′ cap structure also proved to be critical in achieving therapeutic potency [19,20,21,22]. (2) The vast majority of directly injectable mRNA vaccines have a component that protects mRNA from extracellular RNases, facilitates cellular uptake, and often serves as an adjuvant to improve immune responses. Lipid nanoparticles (LNPs) are probably the most frequently used mRNA carriers, but several natural and synthetic polymers have also proved to be efficacious mRNA delivery tools (reviewed in [23,24]). 

Various formats of RNA vaccines against influenza virus have demonstrated potent immunogenicity in preclinical models (Table 1 and discussed in Section 2). Moreover, RNA-based influenza vaccines offer critical advantages over other approaches (detailed in Table 2) such as a favorable safety profile (RNA is a non-infectious, non-integrating molecule degraded by normal cellular processes), highly controllable immunogen production, the absence of anti-vector immunity that enables repeated administration, and, importantly, rapid, scalable production without the use of eggs or complex cell culture systems. The latter is particularly important because some viruses do not grow well in eggs or they can develop egg-adaptive mutations that can alter the antigenicity of the viral surface proteins [25,26,27]. Additionally, generation of FDA-approved conventional influenza virus vaccines can take several months, which can be too long to influence the outcome of an influenza virus pandemic [28]. In contrast, once the genetic sequences of the circulating influenza virus strains are known, RNA vaccines can be easily updated giving an adequate response to viral antigenic drift.

## 2. RNA Vaccines Against Influenza Virus—Vaccine Types, Preclinical and Clinical Data

Influenza virus infection is a major public health problem; thus, tremendous effort has been invested in improving current vaccines and creating new, more effective vaccines over the past decades. The need for a broadly protective/universal influenza virus vaccine, combined with the enormous infrastructure and research experience behind influenza virus research, and the relative ease of testing influenza virus vaccine efficacy in small animal models, have made it an optimal system for RNA vaccine development. Thus, influenza virus RNA vaccines (using IVT RNA) are among the most extensively studied infectious disease RNA vaccines, with studies dating back over 25 years (Table 1). Two major types of RNA vaccines have been developed against influenza virus: self-amplifying mRNA (SAM) vaccines and non-replicating mRNA vaccines (Table 3). SAM vaccines do not use modified nucleosides, while non-replicating mRNA vaccines can be made with or without the incorporation of various modified nucleosides. A great variety of lipid/carbohydrate-based carrier molecules have also been developed that enable highly efficacious in vivo RNA delivery.

### 2.1. Self-Amplifying Influenza Virus RNA Vaccines

The majority of current SAM vaccines use a modified alphavirus genome that encodes the genes for the RNA replication machinery and the antigen of interest in the place of the viral structural protein-encoding genes [35,36]. The full-length RNA is ~9 kilobases long and can be synthetized by IVT from a DNA template. Importantly, the SAM platform enables a high level of immunogen production for extended duration from an exceptionally small dose of vaccine due to intracellular replication of the antigen-encoding RNA. An early study demonstrated that immunization with 10 µg of uncomplexed SAM vaccine encoding A/Puerto Rico/8/34 (H1N1) influenza virus hemagglutinin (HA) elicited antibody responses and partial protection from lethal homologous viral challenge in mice [11]. Hekele and colleagues developed SAM vaccines where the A/California/07/2009 (H1N1) or A/Shanghai/2/2013 (H7N9) influenza virus HA-encoding SAM was formulated in LNPs [37]. Small doses (0.1 or 1 µg) of HA SAM-LNPs induced protective levels of hemagglutination inhibition (HAI) titers after two intramuscular injections in mice. Magini and coworkers developed SAM-LNP vaccines encoding for the nucleoprotein (NP) and matrix protein 1 (M1) from the A/Puerto Rico/8/34 influenza virus [29]. Intramuscular administration of 0.1 or 0.2 µg of NP, M1 or combined NP+M1 SAM-LNP vaccine resulted in potent antigen-specific T cell responses and some level of protection from viral challenge. A seminal work demonstrated that intramuscular delivery of A/California/07/2009 HA-encoding SAM in an oil-in-water nanoemulsion elicited protection from homologous and heterologous (A/Puerto Rico/8/34) influenza virus challenge in mice and ferrets [30]. Influenza virus HA and NP replicon RNA complexed with chitosan-containing LNPs or polyethylenimine (PEI) has elicited T and B cell immune responses in mice after subcutaneous delivery [38,39]. Chahal and colleagues developed a delivery platform consisting of a chemically modified, ionizable dendrimer complexed into LNPs [32]. Using this platform, they demonstrated that intramuscular delivery of RNA replicons encoding A/WSN/33 (H1N1) influenza virus HA protected mice against lethal homologous virus challenge. In a recent study, Vogel and coworkers directly compared the immune responses and protective efficacy after SAM and non-replicating mRNA immunization in mice [31]. Animals were intramuscularly immunized two times with increasing doses of A/Puerto Rico/8/34 HA-encoding unformulated (naked) SAM or unmodified non-replicating mRNA and challenged eight weeks after the first vaccination. Both platforms induced protection against infection with the homologous virus. Of note, 64-fold lower dose (1.25 µg vs. 80 µg) of SAM than non-replicating mRNA generated an equal level of protection. Formulation of HA SAM with PEI significantly increased its efficacy and immunization with HA SAM from various influenza strains induced protection from matched viruses in mice. Immunization with a trivalent SAM vaccine containing HAs from A/California/07/2009 (H1N1), A/Hong Kong/1/68 (X31, H3N2) and B/Massachusetts/2/2012 protected animals from matched H1N1 and H3N2 virus infection. Protection from B/Massachusetts/2/2012 influenza virus was not tested because influenza B-specific antibody responses were inconsistent in mice. Finally, the authors demonstrated that a single immunization with 1.5 µg of PEI-formulated A/California/07/2009 HA SAM induced protection from the homologous virus. 

In summary, several proof-of-concept studies have been published about the efficacy of SAM influenza virus vaccines in preclinical models (Table 1), but no published data from human trials using this vaccine format are available to date. 

### 2.2. Non-Replicating Influenza Virus mRNA Vaccines

A substantial amount of research has been invested into the development of directly injectable non-replicating mRNA vaccines. The first study that demonstrated efficacy after vaccination with mRNA was published in 1993. This seminal work showed that administration of influenza virus NP-encoding mRNA complexed in liposomes induced cytotoxic T cell responses in mice [7]. Interestingly, the first publication that demonstrated protection from influenza virus challenge after mRNA vaccine delivery was published almost 20 years later [33]. Petsch and colleagues intradermally immunized mice, ferrets and pigs with various influenza virus HA, NP and NA-encoding RNActive vaccines (containing unmodified, purified, antigen-encoding and protamine-complexed mRNA [40]) that elicited protective immune responses even after a single immunization. The RNActive vaccine platform has demonstrated potency against various infectious pathogens in multiple preclinical models [33,41]. Kranz and colleagues took an unusual approach and performed intravenous immunizations in mice using A/Puerto Rico/8/34 influenza virus HA-encoding unmodified mRNA-lipid complexes and showed evidence of T cell activation after administration of a single dose [42].

Recently published, highly efficacious influenza virus mRNA vaccines use unmodified or nucleoside-modified full-length HA-encoding mRNAs encapsulated in a LNP carrier. The LNP component of the vaccine has previously been shown to be efficacious for siRNA delivery [43]. CureVac AG developed a sequence-engineered mRNA-LNP platform that enables a high level of in vivo protein translation without the use of modified nucleosides [44]. Using this platform, Lutz and colleagues demonstrated that a single intramuscular immunization with 10 µg of A/Netherlands/602/2009 (H1N1) HA-encoding mRNA-LNPs induced HAI titers in the protective range (≥1:40) in non-human primates (NHPs) [45]. Administration of a second dose potently boosted immune responses and resulted in HAI titers ≥1:160 for over a year in all vaccinated animals. Finally, they provided evidence that two immunizations (4 weeks apart) with mRNA-LNPs encoding for HA from A/Hong Kong/4801/2014 (H3N2) induced stronger T and B cell immune responses than the licensed MF59-adjuvanted trivalent inactivated influenza virus vaccine, Fluad.

Nucleoside-modified mRNA-LNP vaccines represent a new and highly efficacious category of RNA vaccines that can elicit protective immune responses against various pathogens [34,46,47,48]. Several recently published studies provided some insights into the mechanism of action of the nucleoside-modified mRNA-LNP vaccine platform and demonstrated its great ability to create a favorable cytokine/chemokine milieu that rapidly activates critical immune cells and induces potent immune responses after intramuscular or intradermal delivery [49,50,51]. A seminal study from Bahl and colleagues provided evidence that a single intradermal immunization with 10 µg of nucleoside-modified mRNA-LNPs encoding for HA from A/Jiangxi-Donghu/346/2013 (H10N8) or A/Anhui/1/2013 (H7N9) influenza viruses induced durable (>1 year) antibody responses in mice [34]. Furthermore, they showed that administration of a single dose of 0.4 µg of H7N9 HA mRNA-LNPs protected mice from a homologous lethal virus challenge and 10 µg of vaccine reduced lung viral titers in ferrets [34]. Both the H7 and H10 vaccine induced very high HAI titers (~1:10,000) in NHPs after two intradermal or intramuscular immunizations (three weeks apart) with 200–400 µg doses. Due to the promising preclinical results, a Phase I clinical trial (NCT03076385) using this platform was started and the interim analysis of a small cohort of subjects who received two intramuscular doses of LNP-formulated H10N8 HA-encoding nucleoside-modified mRNA was reported [34]. Thirty-one subjects were randomized into groups of 23 vaccinees and 8 saline placebo recipients. The vaccine regimen was 100 µg of mRNA-LNPs administered on day 1 and day 22 (same arm). The study is ongoing and will include a one-year post-vaccination follow up for safety and immunogenicity. In the interim analysis, 43 days after vaccination, no serious systemic adverse events were observed. Two of the subjects who received the vaccine had local reactions that were rated severe (erythema/induration >10 cm in diameter), while none were observed in the placebo group. Systemic reactogenicity was comparable in the vaccinee and placebo groups. Seroprotection (defined as percent of subjects that achieve a HAI titer ≥40 and MNT ≥20) was 100% by HAI and 87% by microneutralization titer (MNT) at day 43. Seroconversion by HAI was 78% (defined as percent of subjects with HAI baseline <10 and post-vaccination HAI ≥40) or 87% (defined as four-fold increase). The geometric mean HAI and MNT titers were increased by 10.6-fold and 7.7-fold, respectively. These results are encouraging in that a microgram-dose vaccine was consistently immunogenic and had an acceptable safety profile similar to other adjuvanted vaccines. Additionally, it is important to note that induction of antibodies for avian strains has proven to be very challenging in clinical trials with traditional protein antigen based vaccines as naïve individuals respond poorly to split virus vaccines containing H5 or H7 HAs [52,53,54].

## 3. Industrial Development of Influenza Virus RNA Vaccines—Global Players in the Field

RNA vaccines have recently received significant attention and attracted massive academic and industrial investment. A limited number of medium-size biotechnology companies have successfully raised capital to develop innovative RNA vaccines (Table 4 and discussed in [10]). Additionally, multiple Phase I and Phase II clinical trials with various RNA vaccines have been conducted to target cancer and infectious pathogens [10]. The first directly injectable infectious disease RNA vaccines that entered clinical trials were against rabies, Zika and influenza viruses [34,48,55]. Several other RNA vaccines are in preclinical development against a wide spectrum of pathogens, including HIV-1, RSV, HCMV and *Coxiella burnetii* [10,56]. Initiation of new clinical trials—including trials targeting influenza virus—is underway. 

Currently, there is one ongoing influenza virus RNA vaccine trial reported on clinicaltrials.gov (NCT03076385) conducted by Moderna Therapeutics (Cambridge, MA, USA). Early results from this Phase I trial using nucleoside-modified mRNA-LNPs are discussed in Section 2.2. CureVac AG (Tübingen, Germany) has plans to initiate its first human seasonal influenza virus RNA vaccine trial, using unmodified mRNA-LNPs, in 2018. Moreover, they announced that an RNActive prophylactic vaccine for seasonal influenza virus will also be evaluated in a Phase I clinical trial in 2018 (www.curevac.com). Both CureVac and Moderna have invested in internal production capabilities. Expected in 2018, CureVac’s “GMP III” facility will have the capacity to produce 10 million doses of GMP mRNA per year, while the commercial scale “GMP IV” facility is currently under construction and will be able to produce up to 30 million mRNA doses per year (www.curevac.com). The company envisions a “GMP V” facility that will be able to increase the capacity to 400 million doses per year (presented at the 5th mRNA Health Conference, Berlin, Germany). Other companies have also announced investments in developing RNA-based influenza virus vaccines; however, little is currently known about their research strategies (Table 4).

## 4. Considerations for Developing a Highly Effective Influenza Virus RNA Vaccine

Companies focused on developing RNA-based influenza vaccines aim to create a product that is superior to the currently available split (inactivated) or LAIV vaccines. Requirements for innovative, broadly protective influenza virus vaccines have been extensively discussed in the influenza vaccine scientific community [57] and the World Health Organization (WHO) has summarized the guidelines in a recent publication, titled Preferred Product Characteristics (PPCs) for Next-Generation Influenza Vaccine [58]. PPCs describe WHO’s preferences for vaccine parameters, including the indications, target groups, immunization strategies, and clinical data for assessment of safety and efficacy. PPCs provide early guidance for the improvement of current influenza virus vaccines and the development of new vaccines, with five and ten-year time horizons (Table 5). The Target Product Profile (TPP) for each RNA vaccine should reflect these PPCs so that the corresponding development plan will meaningfully demonstrate the vaccine candidate’s superior value. The PPC advisory group defined two strategic goals and preferred vaccine characteristics based on WHO’s evaluation of the possible impact on public health. The first goal includes incremental vaccine improvements, such as greater protection against vaccine-matched or drifted influenza virus strains. The second goal aims at greater research and development advances towards vaccines providing broader protection against influenza virus disease (protection across groups or universal influenza virus vaccines) and increased duration of protection (at least five years).

Currently used seasonal influenza vaccines show some level of protection against matched viruses but they offer little protection against drifted seasonal or pandemic viruses. A critical requirement for a new class of vaccine is to greatly improve efficacy and breadth of protection. Prophylactic RNA vaccines demonstrated heterologous protection in preclinical models (Table 1), but translation of this benefit to humans is uncertain and needs to be demonstrated with clinical data. Additionally, individual variations in immune responses to RNA vaccines need to be carefully investigated. The only candidate that has entered clinical development induced protective levels of HAI titers against the homologous virus strain [34], but evaluation of broad protection will require multi-year clinical-endpoint trials. A potential requirement for licensure of a novel RNA influenza vaccine could be the ability to prevent influenza virus disease over several years regardless of antigenic drift in a large-scale field trial. To demonstrate superiority over the conventional vaccine platforms, RNA vaccine candidates will need to be tested in head to head trials, and may be considered to be superior if they protect for longer durations and/or show protection against mismatched strains.

Large-scale efficacy trials are expensive, and thus, an early indication of the level of protection and cross-protection against drifted strains could be provided by Controlled Human Influenza Virus Infection Models (CHIVIM). The utility of such a challenge system for predicting vaccine effectiveness depends on the nature of the challenge virus, its infectious dose, and route of administration. Currently available challenge viruses (influenza H1N1 [59,60] and H3N2 [61]) were isolated four to eight years ago; therefore, finding highly susceptible adult subjects is difficult because of prior exposure. Moreover, the challenge virus dose is large (e.g., 10^5–7^ PFU or TCID_50_) and it is in a liquid suspension or large droplet spray that induce only an upper respiratory tract infection after administration into the nares [59,60,61]. This does not model the typical natural infection, in which an inhaled virus inoculum reaches the upper and lower respiratory tracts. These attributes of the current CHIVIM limit the generalizability of challenge trial outcomes, and restrict its suitability for testing broadly protective influenza countermeasures that are now entering early phase clinical development. Improved CHIVIM are needed to support the development of broadly protective influenza vaccines, including potent RNA vaccines. Ideal CHIVIM would use influenza virus strains to which young adults are susceptible and a suitable but low challenge dose to better mimic natural exposure. 

Immune correlates of protection are invaluable in vaccine development as they minimize complexity, size and cost of clinical trials. While HAI titers are used as a correlate of protection against laboratory-confirmed influenza viruses, no correlates are available for severe disease outcome (or even for lower respiratory tract infection). Non-HA based vaccines or LAIV do not have a known correlate and need to rely on large-scale field efficacy trials. The most potent RNA vaccines induce very high HAI titers in small and large animals and HAI titers in the protective range in humans (discussed in Section 2). Some RNA vaccines have been shown to increase survival in a lethal challenge preclinical model, even in the absence of protective antibodies [34], which suggests that both the cellular and humoral immune responses contribute to RNA vaccine protection. CHIVIM that induce lower respiratory tract disease could be used to explore the correlation of pre-challenge immune measures with reduced risk of illness. This could be an approach to reduce the risk of large trials, and perhaps a pathway for accelerated approval of improved influenza vaccines. 

Durability of protective immunity is also a critical consideration for influenza vaccines. The results of non-human primate studies [34,45] raise the possibility that RNA-based influenza vaccines may elicit more durable immune responses in humans compared to split virus or recombinant protein vaccines. Clinical studies have demonstrated that when naïve subjects are immunized with a vaccine that induces potent cellular immune responses such as LAIV, antibody titers could be boosted with an inactivated virus vaccine up to five years after the original LAIV prime [62]. Since some RNA vaccines have been shown to confer protection before antibodies reached protective titers [34], it would be tempting to test if prime-boost regimens using RNA as a prime would give similar results in preclinical models and humans.

The ultimate goal of an improved and broadly protective influenza virus vaccine is the prevention of influenza virus disease, especially among infants who are at increased risk for severe disease because their respiratory system is immature and they have little to no anti-influenza virus immune memory. This implies that improved vaccines should be indicated for children soon after birth, or not later than three to four months of age if maternal immunization is used to provide passive protection for the first three to four months of life. The lack of an indication for LAIV below two years of age due to increased risk of hospitalization and wheezing is a major limitation of LAIV [63]. RNA vaccines could potentially be evaluated in this patient population, particularly if they are shown to be well-tolerated with an acceptable safety profile in children age two and older. Of note, if RNA vaccines proved to be superior to currently licensed vaccines, a benefit-risk balance could be favorable despite some level of adverse events.

A high level of safety is a critical requirement for vaccines, particularly for those that are administered prophylactically to healthy individuals. RNA vaccine production does not require toxic materials or cell cultures that could be contaminated with viruses; thus, it circumvents the major risk factors associated with manufacturing of live virus, inactivated virus, protein subunit or viral vector-based platforms. Additionally, RNA does not have the ability to integrate into the host cell DNA, thus avoiding the risk of insertional mutagenesis. Potential adverse events such as fever can arise from the potent induction of type I interferons and proinflammatory cytokines by some RNA vaccines [64,65]. Moreover, the presence of extracellular RNA following vaccine administration could potentially raise safety concerns by contributing to formation of pathological thrombus or oedema [66,67]. Although various mRNA vaccine formats have proved to be safe and well tolerated in clinical trials (reviewed in [10]), continuous evaluation of safety of this new platform is critically important.

Due to the limitations of egg-based influenza vaccine production, new manufacturing technologies that do not use eggs to grow viruses have been actively investigated [68]. Vaccines that are produced in cell lines have several advantages, such as a lower risk of adverse events after vaccine administration to people with egg allergies, a sterile manufacturing process that eliminates the use of antibiotics, the absence of egg-adapted mutations, and most importantly, a potentially higher effectiveness [68]. Egg-adapted mutations in the sequence of HA have been associated with low vaccine effectiveness during the 2016–17 influenza season in the United States [26,27]. Zost and colleagues demonstrated that the loss of a glycosylation site by a mutation in the HA of the egg-adapted H3N2 vaccine strain resulted in poor neutralization of the circulating H3N2 viruses in vaccinated humans and ferrets [26]. On the contrary, H3 antigens expressed in the baculovirus-insect cell system did not contain the mutation, and, therefore humans vaccinated with these antigens generated potent neutralizing antibodies against the circulating H3N2 virus [26]. While recombinant protein production by the baculovirus-insect cell system has several critical advantages over egg-based vaccine production, improper glycosylation of HA antigens made by insect cells can be a potential limitation of this approach as reported in a recent study [69]. Vaccine production in mammalian cell lines such as Madin-Darby Canine Kidney (MDCK) cells or Vero cells resolves potential glycosylation issues and allows the production of large amounts of influenza vaccines under carefully controlled conditions, but they require complex and expensive infrastructure [68]. As a comparison, RNA vaccines are produced without the use of eggs or cell culture systems and properly folded and glycosylated mRNA-encoded proteins (vaccine antigens) are made by the host cells after vaccine administration, thus avoiding the risk of the production of incorrect antigens. 

Finally, additional characteristics for a potent RNA vaccine would include considerations on WHO programmatic suitability. Optimal presentation, packaging, thermostability, formulation and disposal are some of the parameters that need to be achieved. WHO has published several documents on programmatic suitability [70] and these requirements should be considered early on during development. In principle, there are no barriers for an RNA influenza vaccine to meet these criteria, and could even be superior to existing vaccines, if, for example, needle-free administration and vaccine stability at ambient temperature could be achieved.

## 5. Conclusions and Future Directions

The past six to eight years brought a clear breakthrough in the fields of cancer and infectious disease RNA vaccines, demonstrating proof-of-concept in both preclinical and clinical settings [10]. Influenza virus RNA vaccines comprise the best-studied RNA vaccines to date. As discussed above, multiple vaccine formats have elicited potent influenza-virus specific, protective immune responses in various preclinical models (Table 1). 

One of the biggest uncertainties of the field is the translatability of the promising animal data to humans. Encouraging early results from the first influenza virus RNA vaccine trial have been published [34]. Long term safety and immunogenicity data from this and other future trials are required to confidently judge the impact of RNA vaccines on the influenza virus vaccine field; thus the coming years will be critical for this new vaccine approach. The authors are optimistic and believe that one or more influenza virus RNA vaccine platform(s) will enter the clinic; however, it is possible that further significant optimization of the current vaccines will be required to decrease the cost of production and increase potency and safety.

There are multiple ways to improve the current influenza virus RNA vaccines. Here, we consider some of the possibilities. As discussed above, most influenza virus RNA vaccine studies used a single full-length HA as an immunogen. Immunization with antibody-accessible conserved influenza virus proteins such as the stalk domain of HA, various domains of NA, and the ectodomain of matrix protein 2 (M2e) has been shown to correlate well with protection in preclinical [71,72] and clinical settings [72,73]; thus it would be intriguing to include these antigens encoded as RNAs in multivalent RNA vaccines. Similarly to the conventional tri- and quadrivalent vaccines, RNA-encoded HAs from various antigenically distant influenza virus strains could be included in a single vaccine regimen to increase neutralization breadth (similarly to the recent study by Vogel and colleagues [31]). In fact, multivalency of RNA vaccines could be easily increased due to the simple manufacturing process and the same supply chain for each coding sequence. The use of optimized RNA-encoded influenza virus immunogens or more efficient immunization schemes (prime-boost) that can elicit favorable immune responses could also lead to more protective vaccines. Finally, addition of various adjuvants (traditional small molecules or RNAs encoding immune modulatory proteins) to RNA vaccines could also increase efficacy. 

As discussed in several recent publications [4,57], an optimal broadly protective/universal influenza vaccine would confer durable protection from various antigenically distant (for example group 1 and group 2 influenza A and influenza B) viruses without causing severe adverse events after vaccine administration. Some RNA vaccines elicit durable influenza virus-specific immune responses in preclinical models, including non-human primates [34,45], and induce protection from heterologous influenza viruses [29,30,33] but protective efficacy across groups has not yet been reported. The production time of broadly protective/universal influenza vaccines should be relatively short (several weeks) which would allow for the protection of the majority of the population from disease caused by a newly emerging seasonal or pandemic virus. The flexibility of RNA vaccine immunogen design and short production time (without the use of eggs and cell culture) are critical advantages over currently approved influenza vaccines. Additionally, storage of all licensed influenza vaccines requires cold chain, while RNActive vaccines have been reported to be active after lyophilization and storage at 5–25 °C for 3 years and at 40 °C for six months [55] and development of mRNA-LNP vaccines that are stable at ambient temperature is underway (Arbutus Biopharma, personal communications).

Collectively, RNA-based vaccines represent a new vaccine class that can be used to effectively combat influenza virus. As noted, more data from clinical trials, including improved controlled human influenza virus infection models, and large-scale field efficacy trials will be critical to demonstrate the viability of this vaccine technology.

## Figures and Tables

**Table 1:** RNA vaccines against influenza virus.*

Vaccine Platform	Immunogens and Route (s) of Administration	Species	Results and References
SAM (uncomplexed)	HA from A/Puerto Rico/8/34; i.m.	mouse	Partial protection from homologous virus [11]
SAM-LNP	NP and M1 from A/Puerto Rico/8/34; i.m.	mouse	Increased survival after homologous and heterosubtypic virus infection [29]
SAM-CNE	HA from A/California/7/2009; i.m.	mouse ferret	Increased survival after homologous and heterologous virus infection [30]
SAM-PEI and unmodified, uncomplexed mRNA	HA from A/California/07/2009, A/Hong Kong/1/68, B/Massachusetts/2/2012; i.m.	mouse	Protection from the homologous viruses [31]
SAM-MDNP	HA from A/WSN/33; i.m.	mouse	Protection from the homologous virus [32]
RNActive vaccine	HA from multiple antigenically distant influenza virus strains, NA and NP from A/Puerto Rico/8/34; i.d.	mouse ferret pig	Protection from homologous viruses and increased survival after heterologous virus challenge in mice, protection from heterologous virus in pigs [33]
Nucleoside-modified mRNA-LNP	HA from A/Jiangxi-Donghu/346/2013 (H10N8) and A/Anhui/1/2013 (H7N9); i.d., i.m.	mouse ferret NHP human	Protection from homologous virus in mice and ferrets, protective HAI titers in NHPs and humans [34]

* only studies where protective efficacy was reported are listed here. SAM: self-amplifying mRNA, LNP: lipid nanoparticle, CNE: cationic nanoemulsion, PEI: polyethylenimine, MDNP: modified dendrimer nanoparticle, NHP: non-human primate, HA: hemagglutinin, NP: nucleoprotein, NA: neuraminidase, M1: matrix protein 1, i.d.: intradermal, i.m.: intramuscular.

**Table 2:** Main characteristics of various influenza vaccine platforms.

Vaccine Platform against Influenza Virus	Safety	Efficacy	Manufacturing
mRNA vaccine	No risks of infection or integration of the vector. Controllable in vivo activity and degradation of mRNA by natural cellular processes. More human data is required to evaluate safety.	Limited efficacy data are available from clinical trials. mRNA vaccines induce immunological correlates of protection and protective effects similar or superior compared to licensed influenza vaccines in preclinical models.	mRNA vaccines are in vitro transcribed in a sterile process that does not require cell culture. The production time is short, the process is sequence-independent and potentially inexpensive and has been demonstrated to be scalable.
DNA vaccine	Good safety record in human studies. Theoretical risks of integration of the vector. Unable to revert to a pathogenic form.	Poor immunogenicity in humans when compared with traditional protein-based vaccines. Ability to induce both humoral and cellular responses. Provide immune priming but poor immune boosting.	Relatively inexpensive. Reproducible, large-scale production. Highly stable vaccines, and no cold chain is required. The production time is short, the process is sequence independent.
Virus-like particle	Influenza vaccines are in clinical development. Licensed vaccines exist for other targets (HBV, HPV) with excellent safety profile.	High effectiveness, and has the ability to induce long-lasting antibody responses.	The major challenge is to develop novel production platforms that overcome issues with current production systems to enable higher throughput at lower cost.
Inactivated virus vaccine	May require adjuvants (for example vaccines for avian strains) that can cause significant reactogenicity.	Cell-based inactivated vaccines are effective for seasonal strains. Pandemic vaccines require use of adjuvants. Good serum antibody responses, but less efficient in triggering mucosal IgA antibodies.	Currently, egg-derived vaccines are the most common in the influenza vaccine market. Cell-based vaccines have demonstrated improved immunogenicity against circulating strains, but manufacturing is challenging and expensive.
Live attenuated influenza virus vaccine	Theoretical risk of recombination with circulating wild-type influenza viruses. Risks of hospitalization and wheezing were increased in children younger than 2 years of age.	LAIV has the ability to induce both humoral and cellular responses. It provides immune priming but low antibody titers.	Only egg-derived vaccines are licensed for use in humans. Cell-based vaccine technologies are under development.

LAIV: live attenuated influenza vaccine

**Table 3:** Advantages and disadvantages of non-replicating mRNA and SAM vaccines.

mRNA Vaccine Platform against Influenza Virus	Potency	Safety	Immunity against the Vector
Non-replicating mRNA	High level of protein translation requires a very efficient delivery system and relatively high doses.	Potent type I interferon response elicited by non-purified and unmodified mRNA can induce serious inflammation. Potential toxic effects may originate from the use of non-natural nucleotides and various delivery system components.	No theoretical risk of anti-vector immunity with non-viral delivery systems.
Self-amplifying mRNA	The auto-replicative ability of SAM enables the production of high levels of vaccine antigen in the host cells. Duration of protein expression from SAM molecules is enhanced.	Similarly to non-replicating unmodified and non-purified mRNA, SAM can induce high level of inflammation. Additionally, SAM-transfected cells likely die due to the continuous replication cycles. Use of a lower effective dose may be possible for SAM compared to non-replicating mRNA.	No anti-vector effect has been observed yet, but potential interactions between encoded non-structural proteins and host factors require additional investigation.

**Table 4:** Companies involved in RNA-based influenza virus vaccine development.

Company Name	Technology/Vaccine Platform	Development Phase
CureVac AG	Sequence-optimized, purified unmodified mRNA (RNActive, RNArt, RNAdjuvant)	Preclinical
Moderna Therapeutics	Nucleoside-modified mRNA	Phase 1
BioNTech Pharmaceuticals	Unspecified	Preclinical
eTheRNA Immunotherapies	Injectable TriMix-mRNA product	Preclinical
Vir Biotechnology	Unspecified	Unknown
EpiVax	T cell epitope vaccine	Preclinical

**Table 5:** Summary of the WHO PPC document on next-generation influenza vaccines and relevant considerations for RNA vaccines.

Preferred Characteristics	Goal 1 (2022)	Goal 2 (2027)	RNA Vaccines
Indication	Prevention of severe influenza illness.	Prevention of severe laboratory-confirmed influenza illness caused by human influenza A virus infection.	Prevention of severe laboratory-confirmed influenza illness caused by human influenza A and B virus infection.
Target population	Children aged 6 weeks through 59 months.	Persons aged 6 weeks and older belonging to a group at high risk for severe influenza illness.	Age de-escalation studies need to be performed to demonstrate safety (for RNA and the formulating agent) in very young children.
Safety	Low level of reactogenicity may be acceptable if the vaccine prevents severe influenza illness.		Low level of reactogenicity can be accepted based on efficacy. Initial studies in humans have shown some reactogenicity.
Co-administration	Documented absence of clinically important interference with concomitantly administered vaccines.		Lack of interference with other vaccines must be demonstrated. Potentially achievable with RNA vaccines.
Duration of protection	>1 full year	>5 years	No human data is available but durable immune responses were observed in animal models, including non-human primates.
Outcome measure and efficacy	Better than standard efficacy (severe laboratory-confirmed influenza illness) for matched or drifted strains.	Better than standard efficacy (severe laboratory-confirmed influenza illness) for matched and drifted strains.	Expression of conserved or engineered antigens and potent, durable T and B cell immunity could lead to broadly protective vaccines.
Immunogenicity	Based on correlates of protection (if the correlates of protection against severe laboratory-confirmed influenza illness is identified for a specific class of influenza vaccine).		A correlate of protection for RNA vaccines has not been established yet, but they have the potential to generate superior cellular and humoral immune responses compared to licensed vaccines.
